# Characterizing the Transmission Dynamics and Control of Ebola Virus Disease

**DOI:** 10.1371/journal.pbio.1002057

**Published:** 2015-01-21

**Authors:** Gerardo Chowell, Hiroshi Nishiura

**Affiliations:** 1 School of Public Health, Georgia State University, Atlanta, Georgia, United States of America; 2 Division of International Epidemiology and Population Studies, Fogarty International Center, National Institutes of Health, Bethesda, Maryland, United States of America; 3 School of Human Evolution and Social Change, Arizona State University, Tempe, Arizona, United States of America; 4 Department of Global Health Policy, Graduate School of Medicine, The University of Tokyo, Tokyo, Japan; 5 CREST, Japan Science and Technology Agency, Kawaguchi, Saitama, Japan

## Abstract

Carefully calibrated transmission models have the potential to guide public health officials on the nature and scale of the interventions required to control epidemics. In the context of the ongoing Ebola virus disease (EVD) epidemic in Liberia, Drake and colleagues, in this issue of *PLOS Biology*, employed an elegant modeling approach to capture the distributions of the number of secondary cases that arise in the community and health care settings in the context of changing population behaviors and increasing hospital capacity. Their findings underscore the role of increasing the rate of safe burials and the fractions of infectious individuals who seek hospitalization together with hospital capacity to achieve epidemic control. However, further modeling efforts of EVD transmission and control in West Africa should utilize the spatial-temporal patterns of spread in the region by incorporating spatial heterogeneity in the transmission process. Detailed datasets are urgently needed to characterize temporal changes in population behaviors, contact networks at different spatial scales, population mobility patterns, adherence to infection control measures in hospital settings, and hospitalization and reporting rates.

## Abstract 2

Carefully calibrated transmission models have the potential to guide public health officials on the nature and scale of the interventions required to control the ongoing Ebola virus disease epidemic in West Africa.

Ebola virus disease (EVD) is caused by an RNA virus of the family *Filoviridae* and *genus Ebolavirus*. Five different *Ebolavirus* strains have been identified, namely *Zaire ebolavirus* (EBOV), *Sudan ebolavirus* (SUDV), *Tai Forest ebolavirus* (TAFV), *Bundibugyo ebolavirus* (BDBV), and *Reston ebolavirus* (RESTV). The great majority of past Ebola outbreaks in humans have been linked to three Ebola strains: EBOV, SUDV, and BDBV [1]. The Ebola virus ([EBOV] formerly designated *Zaire ebolavirus*) derived its name from the Ebola River, located near the epicenter of the first outbreak identified in 1976 in Zaire (now the Democratic Republic of Congo). EVD outbreaks among humans have been associated with direct human exposure to fruit bats—the most likely reservoir of the virus—or through contact with intermediate infected hosts, which include gorillas, chimpanzees, and monkeys. Outbreaks have been reported on average every 1.5 years [2]. Past EVD outbreaks have occurred in relatively isolated areas and have been limited in size and duration (Fig. 1). It has been recently estimated that about 22 million people living in areas of Central and West Africa are at risk of EVD [3].

**Figure 1:**
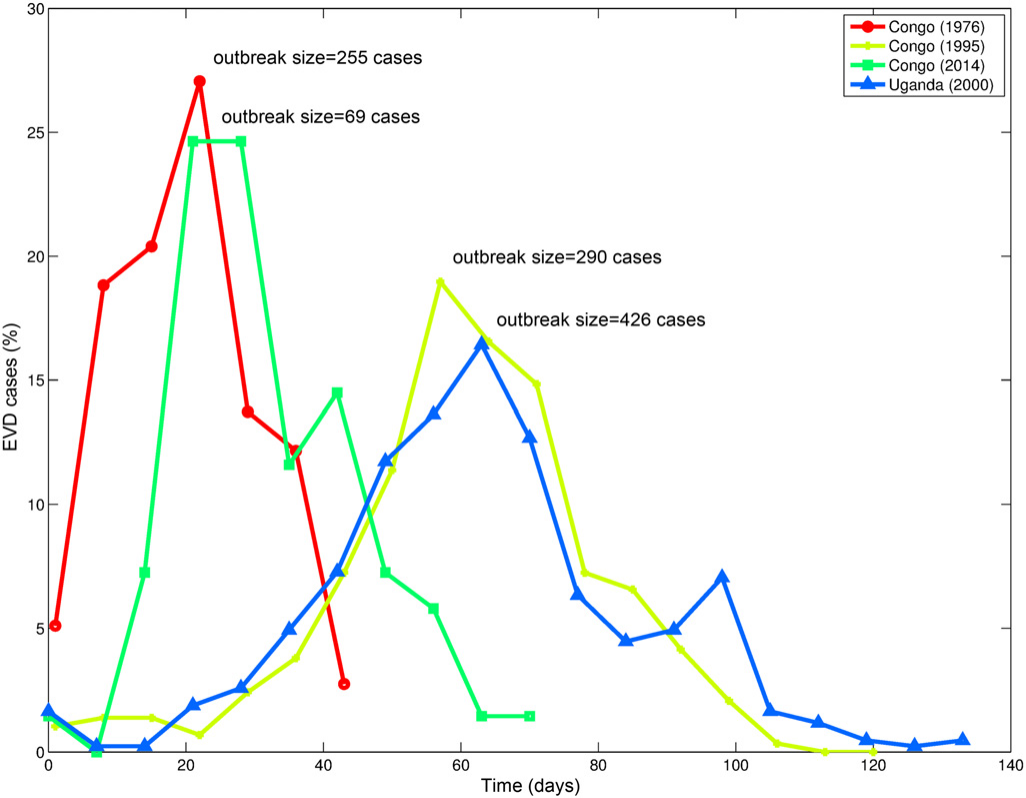
Time series of the temporal progression of four past EVD outbreaks in Congo (1976, 1995, 2014) [4–6] and Uganda (2000) [7]. Case incidence was normalized by the total number of cases reported for each outbreak.

An epidemic of EVD (EBOV) has been spreading in West Africa since December 2013 in Guinea, Liberia, and Sierra Leone [8]. A total of 18,603 cases, with 6,915 deaths, have been reported to the World Health Organization as of December 17, 2014 [9]. While the causative strain associated with this epidemic is closely related to that of past outbreaks in Central Africa [10], three key factors have contributed disproportionately to this unprecedented epidemic: (1) substantial delays in detection and implementation of control efforts in a region characterized by porous borders; (2) limited public health infrastructure including epidemiological surveillance systems and diagnostic testing [11], which are necessary for the timely diagnosis of symptomatic individuals, effective isolation of infectious individuals, contact tracing to rapidly identify new cases, and providing supportive care to increase the chances of survival to EVD infection; and (3) cultural practices that involve touching the body of the deceased and the association of illness with witchcraft or conspiracy theories.

EBOV is transmitted by direct human-to-human contact via body fluids or indirect contact with contaminated surfaces, but it is not spread through the airborne route. Individuals become symptomatic after an average incubation period of 10 days (range 2–21 days) [12], and infectiousness is increased during the later stages of disease [13]. The characteristic symptoms of EVD are nonspecific and include sudden onset of fever, weakness, vomiting, diarrhea, headache, and a sore throat, while only a fraction of the symptomatic individuals present with hemorrhagic manifestations [14]. The case fatality risk (CFR), calculated as the proportion of deaths among the total number of EVD cases with known outcomes, has been estimated from data of the first 9 months of the epidemic in West Africa at 70.8% (95% CI 68.6–72.8), in broad agreement with estimates from past outbreaks [12].

Two important quantities to understand in the transmission dynamics of EVD are the serial interval and the basic reproduction number. The serial interval is defined as the time from illness onset in a primary case to illness onset in a secondary case [15] and has been estimated at 15 days on average for the ongoing epidemic [12]. The basic reproduction number, *R*_0_, quantifies transmission potential at the beginning of an epidemic and is defined as the average number of secondary cases generated by a typical infected individual during the early phase of an epidemic, before interventions are put in place [16]. If *R*_0_ < 1, transmission is not sufficient to generate a major epidemic. In contrast, a major epidemic is likely to occur whenever *R*_0_ > 1. When transmission potential is measured over time *t*, the effective reproduction number *R_t_*, can be helpful to quantify the time-dependent transmission potential resulting from the effect of control interventions and behavior changes [17]. Estimates of *R*_0_ for the ongoing epidemic in West Africa have fluctuated around 2 with some uncertainty (e.g., [12, 18–22]), which are in good agreement with estimates from past EVD outbreaks [23]. *R*_0_ could also vary across regions as a function of the local public health infrastructure (e.g., availability of health care settings and infection control protocols), such that an outbreak may be very unlikely to unfold in developed countries simply as a result of baseline infection control measures in place (i.e., *R*_0_ < 1) while poor countries with extremely weak or absent public health systems may be unable to control an Ebola outbreak (i.e., *R*_0_ > 1).

Mathematical models of disease transmission have proved to be useful tools to characterize the transmission dynamics of infectious diseases and evaluate the effects of control intervention strategies in order to inform public health policy [16, 24, 25]. There are a limited number of mathematical models for the transmission and control of EVD, but a number of efforts are underway in the context of the epidemic in West Africa. The transmission dynamics of EVD have been modeled on the basis of the simple compartmental susceptible-exposed-infectious-removed (SEIR) model that assumes a homogenously mixed population [23]. The modeled population can be structured according to the contributions of community, hospital, and unsafe burials to transmission as EVD transmission has been amplified in health care settings with ineffective infection control measures and during unsafe burials [23]. A schematic representation of the main transmission pathways of EVD is shown in Fig. 2.

**Figure 2:**
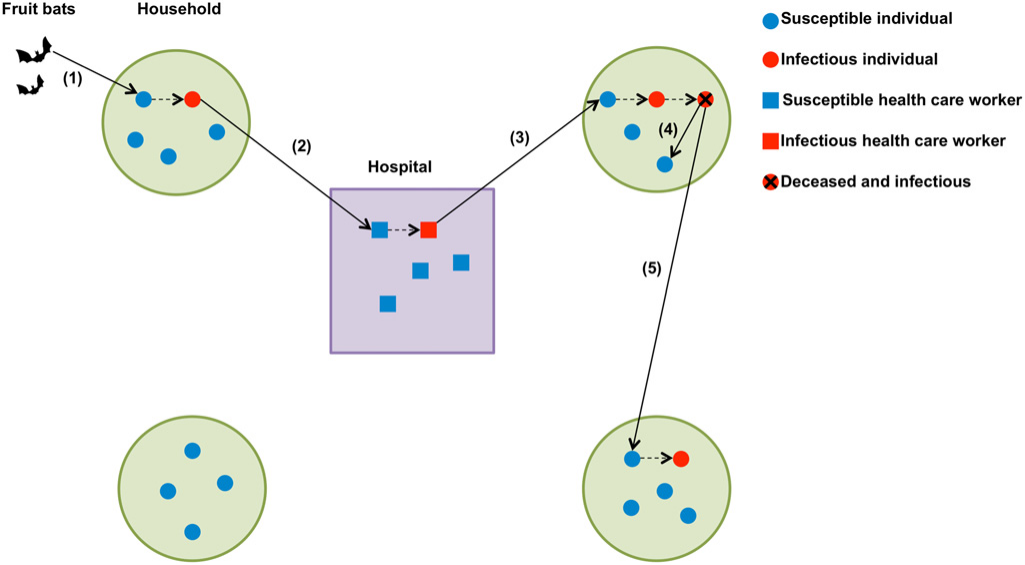
Schematic representation of the transmission dynamics of Ebola virus disease. The main transmission pathways are: (1) Spillover transmission to humans by direct contact with a reservoir (e.g., fruit bats); (2) within hospital transmission from EVD patients to health care workers; (3) within household transmission from infected health care workers to their family members; (4) within household transmission during unsafe burials; and (5) inter-household transmission arising from unsafe burials. Solid arrows indicate a transmission link while dashed arrows denote disease progression (e.g., from susceptible to infectious or from infectious to dead and infectious).

A recent study published in *PLOS Biology* by Drake and colleagues [26] presents an interesting and flexible modeling framework for the transmission and control of EVD in Liberia. Their framework is based on a multi-type branching process model in which “multi-type” refers to the consideration of two types of settings where transmission can occur, while “branching process” is the mathematical term to specify a probabilistic model. For instance, in the case of a single-type branching process, the transmission dynamics are simply described using a single reproduction number, i.e., the average number of secondary cases produced by a single primary case. However, when two types of hosts are considered in the transmission process, two reproduction numbers are needed to characterize within-group mixing (e.g., within-hospital and within-community transmission) and two reproduction numbers characterize transmission between groups (e.g., transmission from hospital to community and vice versa).

Drake and colleague’s elegant modeling approach describes EVD transmission according to infection generations by calculating probability distributions of the number of secondary cases that arise in the community via nursing care or during unsafe burials and in health care settings via infections to health care workers and visitors. The model explicitly accounts for the hospitalization rate—the fraction of infectious individuals in the community seeking hospitalization (estimated in this study at 60%). However, the number of effectively isolated infectious individuals is constrained by the number of available beds in treatment centers—which are assumed in this study to operate at twice their regular capacity. It is important to note that the number of beds available to treat EVD patients was severely limited in Liberia prior to mid August 2014 (Fig. 1 in [26]). Moreover, the rate of safe burials that reduces the force of infection is included in their model as an increasing function of time. The model was calibrated by tuning six parameters to fit the trajectories of the number of reported cases in the community and among health care workers during the period 4 July to 2 September 2014 for a total of four infection generations during which the effective reproduction number was estimated to decline on average from about 2.8 to 1.4. The model was able to effectively capture heterogeneity in transmission of EVD in both the community and hospital settings.

Drake and colleagues [26] employed their calibrated model to forecast the epidemic trajectory in Liberia from 3 September to 31 December 2014 under different scenarios that account for an increasing fraction of cases seeking hospitalization and a surge in the number of beds available to isolate and treat EVD patients. Their results indicate that allocating 1,700 additional beds (100 new beds every 4 days) in new Ebola treatment centers committed by US aid reduces the mean epidemic size to ~51,000 (60% reduction with respect to the baseline scenario), while epidemic control by mid-March is only plausible through a 4-fold increase in the number of beds committed by US aid and enhancing the hospitalization rate from 60% to 99% for a final epidemic size of 12,285. Moreover, an additional epidemic forecast incorporating data up to 1 December 2014 indicated that containment could be achieved between March and June 2015.

Other interventions were not explicitly incorporated in their model because it is difficult to parameterize them in the absence of datasets that permit statistical estimation of their impact on the transmission dynamics. These additional interventions include the use of household protection kits, designed to reduce transmission in the community; improvements in infection control protocols in health care settings that reduce transmission among health care workers; and the impact of rapid diagnostic kits in Ebola treatment centers, which reduce the time to isolation for infectious individuals seeking hospitalization. Increasing awareness and education of the population about the disease could have also yielded further reductions in case incidence by reducing the size of the at-risk susceptible population (Fig. 3) [27]. Nevertheless, some of these effects could have been indirectly captured implicitly by the time-dependent safe burial rate parameter in their model.

**Figure 3:**
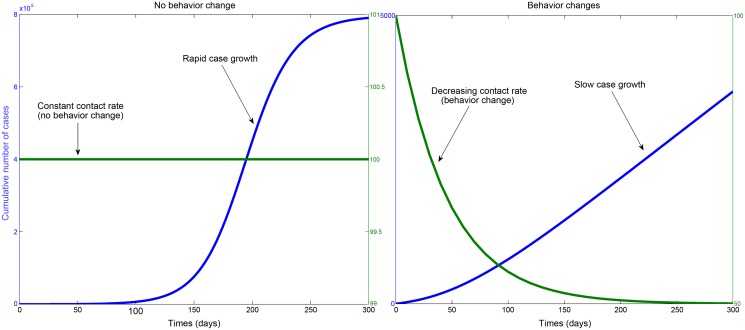
Contrasting epidemic growth in the presence and absence of behavior changes that reduce the transmission rate. In the left panel, the epidemic grows exponentially fast for a constant average contact rate over time (no behavior changes). In the right panel, the population changes behavior as public awareness about the disease increases, leading to a gradually decreasing contact rate. The basic reproduction number *R*_0_ is set at 2.0 for both scenarios with and without behavior changes.

Importantly, prior models of EVD transmission [23, 28, 29] and the model by Drake and colleagues have not incorporated spatial heterogeneity in the transmission dynamics. In particular, the EVD epidemic in West Africa can be characterized as a set of asynchronous local (e.g., district) epidemics that exhibit sub-exponential growth, which could be driven by a highly clustered underlying contact network or population behavior changes induced by the accumulation of morbidity and mortality rates (see Fig. 4 and [30]). EVD contagiousness is most pronounced in the later and more severe stages of Ebola infection when infectious individuals are confined at home or health care settings and mostly exposed to caregivers (e.g., health care workers, family members) [30]. This characterization would lead to EVD transmission over a network of contacts that is highly clustered (e.g., individuals are likely to share a significant fraction of their contacts), which is associated with significantly slower spread relative to the common random mixing assumption as illustrated in Fig. 5. The development of transmission models that incorporate spatial heterogeneity (e.g., by modeling spatial coupling or human migration) is currently limited by the shortage of detailed datasets from the EVD-affected areas about the geographic distribution of households, health care settings, reporting and hospitalization rates across urban and rural areas, and patterns of population mobility in the region. Some of these limitations may be overcome in the near future. For instance, cell phone data could provide a basis to characterize population mobility in the region at a refined spatial scale.

**Figure 4:**
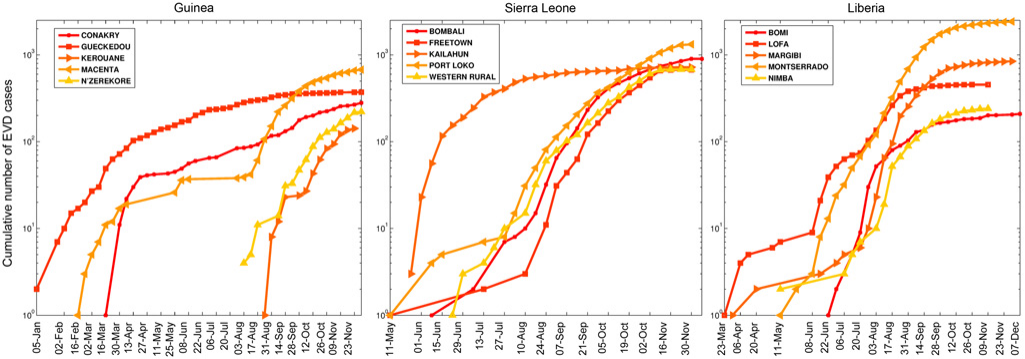
Representative time series of the cumulative number of EVD cases (in log scale) at the district level in Guinea, Sierra Leone, and Liberia. The district level epidemics are asynchronous and exhibit sub-exponential case growth.

**Figure 5:**
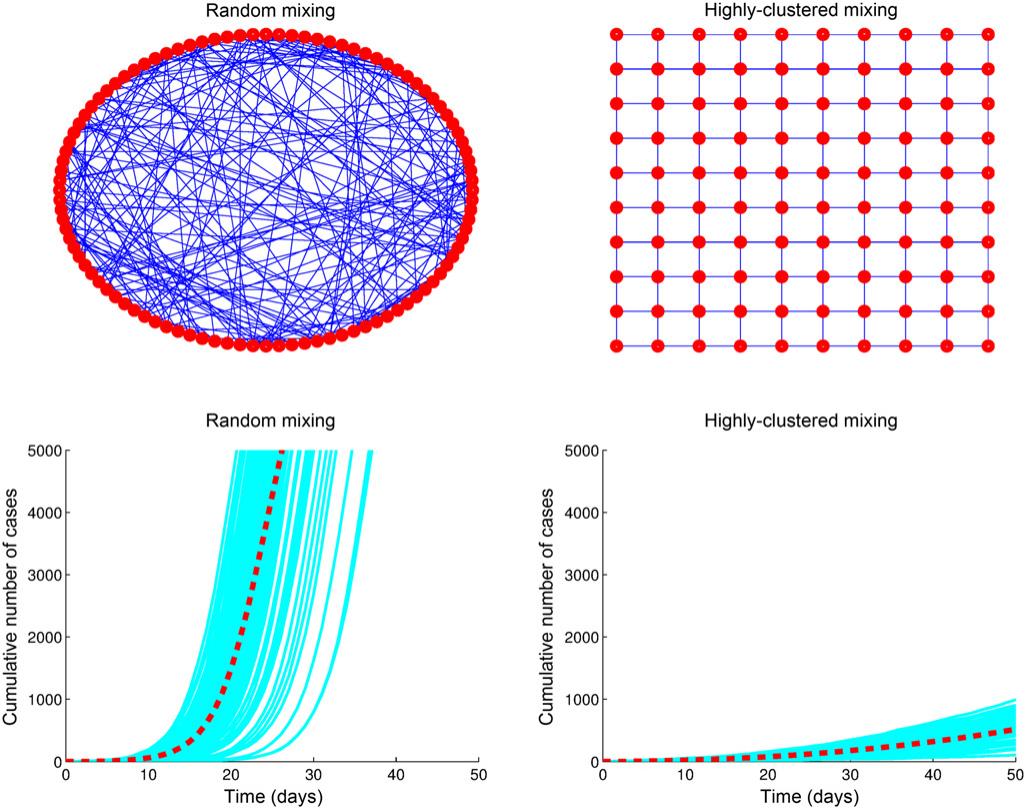
Epidemic growth in two populations characterized by two different underlying contact networks. Random mixing leads to a rapidly disseminating infectious disease that spreads exponentially fast. By contrast, disease spread is significantly slower in a highly clustered contact network because the contacts of recently infected individuals are likely to be already infected by other infectious individuals. The contact networks are shown for illustration and are composed of a set of nodes (individuals) denoted by red dots while blue lines (links) between nodes denote static contacts. Each solid blue curve of cumulative cases corresponds to a stochastic epidemic realization of the simple SEIR (susceptible-exposed-infectious-removed) [16], while the dashed red curve corresponds to the average of 100 stochastic realizations.

The ongoing epidemic in West Africa offers a unique opportunity to improve our current understanding of the transmission characteristics of EVD in humans. To achieve this goal, it is crucial to collect spatial-temporal data on population behaviors, contact networks, social distancing measures, and education campaigns. Datasets comprising detailed demographic, socio-economic, contact rates, and population mobility estimates in the region (e.g., commuting networks, air traffic) need to be integrated and made publicly available in order to develop highly resolved transmission models, which could guide control strategies with greater precision in the context of the EVD epidemic in West Africa. Although recent data from Liberia indicates that the epidemic is on track for eventual control, the epidemic in Sierra Leone continues an increasing trend, and in Guinea, case incidence roughly follows a steady trend. The potential impact of vaccines should also be incorporated in future modeling efforts as these pharmaceutical interventions are expected to become available in the upcoming months.
